# RoMoMatteR: Empowering Roma Girls’ Mattering through Reproductive Justice

**DOI:** 10.3390/ijerph17228498

**Published:** 2020-11-17

**Authors:** Manuel Garcia-Ramirez, Belen Soto-Ponce, María J. Albar-Marín, Daniel La Parra-Casado, Dena Popova, Raluca Tomsa

**Affiliations:** 1 CESPYD, University of Seville, 41018 Seville, Spain; bsponce@us.es (B.S.-P.); mja@us.es (M.J.A.-M.); 2 Department of Sociology 2, University of Alicante, 03690 Alicante, Spain; daniel.laparra@ua.es; 3 TSA, Trust for Social Achievement, 1000 Sofia, Bulgaria; dpopova@tsa-bulgaria.org; 4 Department of Psychology, University of Bucharest, 050663 Bucharest, Romania; raluca.tomsa@fpse.unibuc.ro

**Keywords:** Roma girls, mattering, reproductive justice, community based participatory action research, photovoice

## Abstract

*Aim*: To present a protocol study directed at tackling gender discrimination against Roma girls by empowering their mattering so they can envision their own futures and choose motherhood only if—and when—they are ready. *Background*: Motherhood among Roma girls (RGM) in Europe impoverishes their lives, puts them at risk of poor physical and mental health and precipitates school dropouts. Overwhelming evidence affirms that the conditions of poverty and the social exclusionary processes they suffer have a very important explanatory weight in their sexual and reproductive decisions. *Methods*: Through a Community-based Participatory Action Research design, 20–25 Roma girls will be recruited in each one of the four impoverished communities in Bulgaria, Romania and Spain. *Data collection and analysis*: Desk review about scientific evidences and policies will be carried out to frame the problem. Narratives of Roma women as well as baseline and end line interviews of girl participants will be collected through both qualitative and quantitative techniques. Quantitative data will be gathered through reliable scales of mattering, socio–political agency, satisfaction with life and self. A narrative analysis of the qualitative information generated in the interviews will be carried out. *Expected results*: (1) uncover contextual and psychosocial patterns of girl-motherhood among Roma women; (2) build critical thinking among Roma girls to actively participate in all decisions affecting them and advocate for their own gender rights within their communities; and (3) empower Roma girls and their significant adults to critically evaluate their own initiatives and provide feedback to their relevant stakeholders. *Conclusions*: Roma girls will improve their educational aspirations and achievements and their social status while respecting and enhancing Roma values.

## 1. Introduction

Roma Girls Motherhood (RGM) is deeply embedded in a tangle of multiple discriminations and used by certain sectors of European society to validate the stereotyping and rejection of Roma [1,2,3,4,5]. Research in this area consistently affirms that RGM is one of the factors that impoverishes the life of girls, increases the risks of suffering physical and mental health problems, makes them vulnerable to intimate partner violence, and prevents them from remaining in the educational system and successfully inserting themselves into the labor market [6,7,8]. RGM violates the fundamental right of adolescents to “be girls” and jeopardizes their children’s rights to enjoy parental care that ensures their development [9]. Although ethnicity is not usually collected in the available statistical data, reliable estimations indicated that nearly 2% of European Roma girls between 10 and 15 are traditionally married or cohabit with their partner; only 6% of Roma teenage mothers complete primary schooling and they generally dedicate their time to housekeeping [10,11,12].

### 1.1. Roma Girls’ Mattering, Motherhood and Structural Discriminatory Mechanisms

From a psychosocial point of view, mattering refers the process of development of human beings through which the adolescent “perceives and feels that she counts to others, that others care about her future, and that others depend on her—that they need her” [13] (p. 165). Therefore, mattering implies recognition and influence in the social sphere. Recognition refers to the signs of acceptance that we receive from our significant environment, our family and peers, social referents, and our community. Influence refers to the certainty that what we do is important to others and that others need us to satisfy their needs [14,15,16,17]. Mattering is essential in social development and the development of the self since there are a few things worse than feeling irrelevant to others [13]. Elliot, Kao and Grant argue that child motherhood could be better understood if we explain it as women and girls seeking to be valued by others in settings where they are irrelevant and undervalued [15]. In fact, many studies have shown that child motherhood is often associated with emotional stability, purpose, and responsibility as well as a strong sense of belonging to a cultural community [7,18,19]. This is especially explanatory in impoverished Roma communities where girls happily assume motherhood in return for acquiring mattering despite the hard limitations entailed in their lives and without questioning why their parents and close relatives promote it despite knowing the limitations this poses in their lives [2,4,20,21]. 

In spite of this evidence, it is common to attribute RGM to “cultural patterns” in which Roma women’s mattering is anchored to the roles of wife, mother, and caregiver [22,23]. This explanation ignores the fact that Roma culture is continually built by Roma people when they use—and update—inherited cultural tools (e.g., language, symbolism, rites, and shared history) in their daily lives (e.g., going to school, being a mother, caring for loved ones) in the context of oppressive ethnic conditions [24,25]. RGM should be explained by highlighting how cultural Roma patterns intersect with oppressive contextual mechanisms (i.e., violence, cultural and sexual control, economic exploitation, and exclusion from the political and social spheres) and condemn women and girls to irrelevance and a lack of agency in their daily lives across their lifetime [25,26,27]. Violence against Roma women is systematic and structural [28]. The services responsible for its prevention and punishment fail in these tasks by questioning the accusations or blaming the victims, thus creating fear, low self-esteem, and defenselessness among Roma girls. Economic exploitation of these women translates into unemployment, precarious or unskilled labor, cheap labor, or the exclusive care of their home or others. Political and social exclusion—often by invisible structural mechanisms—instill shame and fear among Roma women and girls that prevents them from participating and intervening in public spaces where men’s agency and presence is much more evident. In impoverished Roma communities, the power of women is often yielded to the men, first their fathers, then their husbands. This cultural control also entails sexual control that forces Roma girls to submit their bodies—and the decisions about them—to the pressures, demands, and expectations of general population/others in issues like virginity, marriage, or maternity. These discriminatory and oppressive mechanisms smother any opportunity for self-determination, undermining the pillars on which Roma girls construct their gender identity, build their families, choose their partners, and decide on motherhood [2,4]. 

Several institutions, including the Directorate General for Justice of the European Union, are committed to developing a body of shared knowledge to overcome the conditions of inequality and exclusion suffered by Roma girls [29]. These recommendations are consistent with the Guttmacher–Lancet Commission, which recognizes the need for more research on the gender rights of girls aged 10 to 14, with the use of rigorous and innovative methodologies and ethical guarantees [30].

### 1.2. Linking Roma Girls’ Mattering to Reproductive Justice

**Figure 1:**
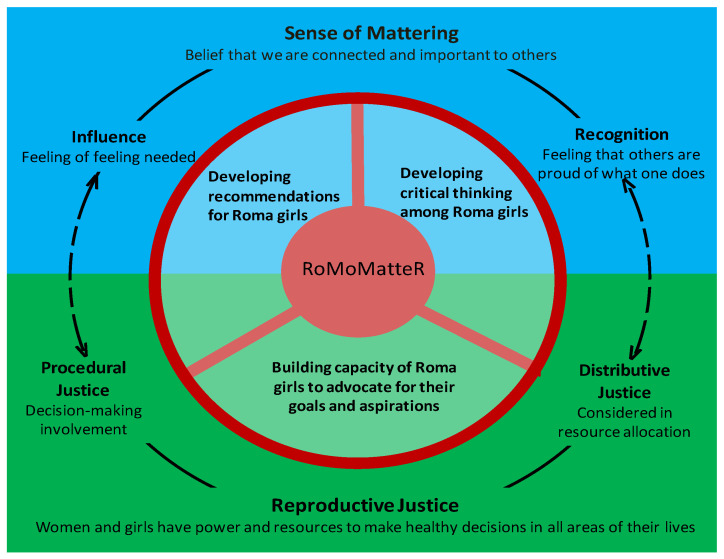
Sense of mattering linked to reproductive justice.

RoMoMatteR (Roma Mom Matter) maintains that the discrimination suffered by the Roma girls could be reversed through a process of analysis and empowerment that links their mattering to reproductive justice (see Figure 1) [26,31,32]. We understand reproductive justice as “the complete physical, mental, spiritual, political, economic, and social well-being of women and girls, which will be achieved when women and girls have the economic, social, and political power and resources to make healthy decisions about [their] bodies, sexuality, and reproduction of ourselves, [their] families, and [their] communities in all areas of [their] lives” [33] (p. 1).

Reproductive justice implies the right to (a) make life decisions without discrimination, coercion, or violence; (b) privacy, confidentiality, respect, and informed consent; and (c) enjoyment of respectful and equitable relationships [30]. Consequently, reproductive justice entails having opportunities to make decisions (i.e., procedural justice) and access to sufficient resources (i.e., distributive justice). The acquisition of recognition linked to distributive justice implies acceptance and respect for Roma girls’ identities, their life goals, and the guarantee of resources to achieve them. The acquisition of influence linked to procedural justice guarantees processes in which Roma girls have their own voice and are recognized as policy agents. 

On the other hand, the development of Roma girls’ mattering linked to reproductive justice involves all relevant domains for their psychosocial development. First, this process involves acquiring critical thinking and knowledge through reflection and evaluation. Critical thinking will entail learning and realizing that their life conditions can change and are not determined by nature. This invitation to agency recognition will allow for new interpretations and narratives on motherhood, mothering, and their cultural values and practices. Second, this process will allow Roma girls to imagine new possibilities for their lives, be aware of new resources that are available, and be open to desire them, set agentic goals and plan the needed steps to achieve them, reflect and learn from mistakes and successes, learn new roles, and build new networks without losing the respect of their communities, relatives, and significant others. Finally, this process will enable them to actively advocate for the construction of safe and healthy contexts for themselves, their children, their loved ones, and their communities [25,34,35]. 

RoMoMatteR assumes that Roma girls have the talent and ability to lead initiatives that confront the challenges that concern them [32,33]. RoMoMatteR will carry out processes based on the collaborative alliance between Roma girls and their significant adults (i.e., parents, public service providers, teachers and other significant adults), creating spaces in which Roma girls will identify their needs and strengths, design actions, implement, evaluate, and provide feedback [34,35]. 

### 1.3. Aims

The goal of RoMoMatteR is to tackle gender discrimination by empowering Roma girls’ mattering to allow them to envision their own futures and build capabilities to advocate for equal opportunities and then, to choose motherhood only if—and when—they are ready. 

Specific aims are:

(1) To uncover contextual and psychosocial patterns of girl-motherhood among Roma women.
(2) To build critical thinking among Roma girls to actively participate in all decisions affecting them and advocate for their own gender rights within their communities.
(3) To empower Roma girls to critically evaluate their own initiatives and provide feedback to their relevant stakeholders.

## 2. Materials and Methods

### 2.1. Design

RoMoMatteR will conduct Community-based Participatory Action Research (CBPAR) processes based on the collaborative alliance between Roma girls and their key stakeholders (i.e., family, public service providers, and other significant adults). Academic researchers act as instigators, mediators, and technical assistants [35,36,37]. CBPAR is the most appropriate methodological strategy for RoMoMatteR because it is guided by girls’ needs and resources, giving them a voice to identify their needs and advocate for their rights. Therefore, CBPAR generates processes that monitor and combat discrimination, racism, and sexism in research/action through ongoing reviews of decision making. 

### 2.2. Settings, Participants and Eligible Criteria

The RoMoMatteR transnational consortium is comprised of Roma communities, civil society groups, local institutions, and scientific centers from Bulgaria, Romania, Spain, Hungary, and the UK. From a convenience sampling, 20–25 Roma girls will be recruited in each one of the four community contexts, i.e., Alicante and Córdoba (Spain), Bucharest (Romania) and Straldzha (Bulgaria); aged between 10 and 14 years old, who identify themselves as Roma, have no children, are not pregnant, attend school, and are of national or foreign origin. 

Moreover, around 65 key stakeholders have been directly involved in project activities: key Roma women, providers of public services (e.g., health, education, social affairs, employment), staff members of community organizations working with Roma populations, and other significant adults identified by girls and their families. Three Roma women from each community context who are not mothers of participant girls and have a good reputation and experience in community work will act as facilitators of Photovoice sessions.

### 2.3. Work Plan

**Figure 2:**
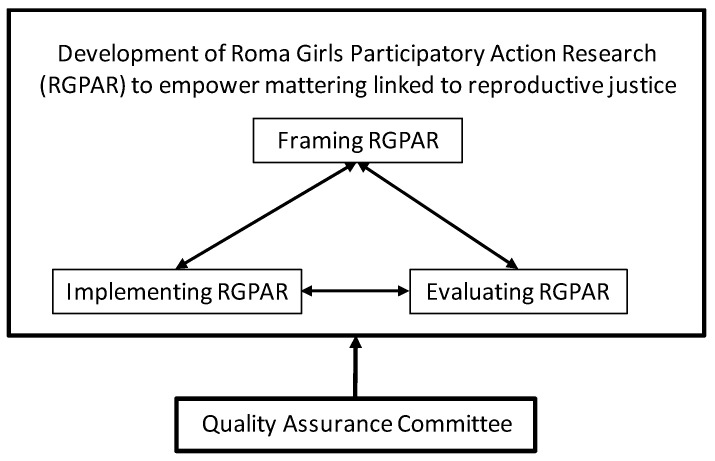
Work plan of the Roma Girls’ Participatory Action-Research.

RoMoMatter will be developed from three work packages—each led by one of the fieldworks’ partnerships of the European consortium; i.e., a community organization and research organization. The University of Seville acts as coordinator, and a Quality Team—composed of international experts—supports the fieldwork teams to ensure the quality of actions. Figure 2 shows the structure of RoMoMatteR and below, the work packages will be described.

#### 2.3.1. Framing Roma Girls’ Participatory Action Research to Empower Mattering Linked to Reproductive Justice

At the beginning of the project, a local coalition will be built in each context to facilitate understanding of cultural groups and create trust and a collaborative environment. Different mappings will be conducted to develop a contextual framework for RGPAR to empower their mattering through reproductive justice. The main deliverables are listed in Table 1.

#### 2.3.2. Implementation of RGPAR: Activities and Procedures

The objective of this phase is to engage Roma girls and their communities in developing critical thinking, building recommendations, and advocating for the empowerment of mattering linked to reproductive justice through Photovoice. Photovoice is recognized as a strong method for including a CBPAR approach [35], to develop a deep understanding of contexts, spaces, constructions, and the role of the community and its resources [35,40].

Roma women facilitators will guide RGPAR processes in each of the 4 local contexts, arranging encounters and interactions with Role Models, encouraging Roma girls’ active involvement, inspiring them to take the role of active researchers and making their voices heard (as photographers, journalists), valuing girls’ different opinions and ideas, and acting as a liaison between Roma girls, the Local Coalition, and communities. They will be accompanied by technical/research assistants throughout the project as well as research and community partners. Table 2 defines the activities and procedures of the different components of this work package.

#### 2.3.3. Building Evaluation Capacity among Girls and their Communities

The research team will implement a participatory evaluation approach that will engage all project stakeholders, with a focus on girls and facilitators as active evaluators of the whole initiative. The evaluation methodology reflects the mapping of community insight and knowledge developed by the consortium and has been designed in close synergy with the RGPAR development. The research team will build evaluation capacity among community partners, coalition members, facilitators and Roma girls to ensure that the agents involved have enough sensitivity, skills, resources, feedback, and advice to carry out their responsibilities. RoMoMatteR uses Empowerment Evaluation (EE) as the methodological approach to achieve this goal. The EE framework was proposed by Fetterman and Bowman, who define it as “the use of evaluation concepts, techniques, and findings to foster improvement and self-determination among communities” [42]. Table 3 shows the design, goals and indicators of RGPAR’s process, implementation, and outcome evaluation. 

There is a combination of process evaluation (ongoing at session-level) and four specific evaluation milestones. After each Photovoice session, the facilitators will meet with the Field Work Group for follow-up, reflection and planning future steps. The four core milestones are: (a) recruitment process (baseline); (b) community exhibition of dreams and aspirations of Roma girls; (c) community exhibition on resources and launch of media advocacy campaigns proposed by girls; (d) end line interviews with all participating agents in the project; i.e., facilitators, girls, families, and community partners.

### 2.4. Data Collection

To frame the RGPAR, multiple techniques of data collection—such as scoping reviews, stakeholder interviews, women’s in-depth interviews, and focus groups will be used. Included databases will be PubMed/MEDLINE, Web of Science, Scopus and PsycINFO using thematic filters related to Roma populations (e.g., Roma, Romani, Gypsy, Gypsies) and sexual and reproductive health (e.g., motherhood, maternity, childbearing, reproductive justice, reproductive rights).

Regarding the process and implementation evaluation activities, information will be gathered through meeting notes, observation forms, attendance sheets, satisfaction forms, emotional climate of sessions, participation/adherence levels for each girl and quality assurance checklists. 

Regarding outcomes assessment, baseline and exit interviews will be carried out among girl participants and their significant adults, including qualitative and quantitative data. Semi-structured interviews will be used to gather qualitative data from Roma girls using a social network scale (Social Convoy [43]). Quantitative data will be gathered through different scales and questionnaires such as mattering scale [15], socio-political agency scale [44]; life satisfaction scale [45] and relevance of identity roles scale among others (see Appendix A). The comparison group will fill in the same scales.

All interviews be carried out by two people in a quiet, calm and private environment (e.g., school, community center). A climate of trust, confidentiality and familiarity will be generated at the beginning of the interview. The interviews should be audio-recorded. One person should conduct the interview and the other one should take notes and be responsible for the audio-recording. The audio-recordings and transcriptions need to be downloaded to password-protected folders with access only by the Research Partner. It is advised that the checklist forms are filled in by both interviewers immediately after the interview, mitigating the risk that the data may be forgotten or mis-recorded.

### 2.5. Data Analysis 

A content analysis of the qualitative information generated in the interviews and focus groups will be carried out using the software Atlas-ti or NVivo. The unit of analysis will be the conducted verbatim transcriptions of interviews, from which thematic categories will be generated and analyzed following Corbin and Strauss’ guidelines [46]. 

To evaluate the effect of the Participatory Action Research intervention, statistical analysis will be performed using IBM SPSS Statistics Version 18.0 (IBM, New York, NY, USA). Descriptive analyses (mean and standard deviations) and two-way repeated-measures ANOVAs will be carried out. Statistical assumptions for parametric tests will be checked. Equivalence between (1) the intervention group and the comparison group and (2) completers and drop-outs will be examined by performing one-way ANOVAs for quantitative variables and χ^2^ test for qualitative variables on socio-demographic characteristics and dependent variables at baseline. Differences in the social networks of participant girls will be obtained through social network analysis.

### 2.6. Quality Assurance Advisory Committee (QAAC)

The QAAC will serve as the authority within the project on issues related to quality assurance during the entire project’s lifetime. Thus, the QAAC’s purpose is to improve the overall quality of the methodology implemented, supervise other relevant partners in the implementation and reflection of activities, deliverables, and results obtained. Among its responsibilities are the development of technical quality control guidelines (Quality assurance plan), the coordination of the development and use of tools to measure data quality, enhancing quality control feedback during project implementation, the delivering of quality assurance reports (four in total), and the design and implementation of a Risk Management Plan. Special attention will be focused on developing a guideline. The QAAC will report directly to the Steering Committee.

More specifically, the tasks of the QAAC are, firstly, to improve the quality of draft texts (e.g., deliverables). The quality of action plans can be assessed, and recommendations for improvement issued. Similarly, the quality of process and implementation can be assessed, and recommendations are promptly acted on. The quality of outcomes can be also assessed (i.e., the extent to which intended outcomes were achieved), but it must be borne in mind that this does not simply reflect how well the work was carried out. An action may fail to achieve its goals not because it was poorly executed, but because its goals were unrealistic in the first place, or because unforeseen factors prevented their achievement.

### 2.7. Resources and Infrastructures

Available community infrastructures will be used (e.g., schools, community organizations, churches, health services, etc.). The involved academic institutions have the software needed for the qualitative analysis of the gathered narratives (i.e., Atlas Ti; NVivo) as well as quantitative data (i.e., SPSS). Other tools to store the information generated during the process, as well as the means needed to process the information produced, will be available to researchers and Roma organizations. The spaces for conducting training sessions and implementation of the project will be provided by schools, Roma organizations, or other community centers.

To implement Photovoice activities, Roma girls will also be provided with cameras and training so that they can become active researchers in the study and be involved in all activities, from data collection to the sharing of results. 

### 2.8. Ethical Considerations

In RoMoMatteR, techniques for collecting quantitative and qualitative data will be applied to under-age pre-adolescents, community services professionals, and families. All members of the research teams of partners will guarantee the anonymity and confidentiality of the information collected. The Commission Recommendation of 17 July 2012 on access to and preservation of scientific information will be met, as well as the EU Directive 95/46/EC of the European Parliament and of the Council of 24 October 1995 on the protection of individuals concerning the processing of personal data and the free movement of such data. Following these regulations, an informed consent document will be drawn up for participants to sign, which will reflect these aspects. In the case of pre-adolescents, we will also have their parents’ consent and the approval of ethics committees of participant universities. 

The informed consent document will include: (a) an explanation of the objectives of the study, its duration, and the time of participation of the subject, who may voluntarily leave the study at any time without any negative repercussions; (b) a statement that participation is voluntary and informed; (c) information on the funding of the research, as well as the guarantee that participation will not imply any expense; (d) a description of the benefits for the subjects or third parties; (e) a statement of procedures to ensure data protection/confidentiality/privacy; (f) the name of the contact person for any questions related to the project and; (g) information on the consequences of the results.

## 3. Discussion

RoMoMatter will empower Roma girls to (a) identify the social determinants and patterns of psychosocial development associated with their reproductive decisions, (b) construct gender-rights-based self-narratives, and (c) advocate socio–political changes that will ensure their well-being and prosperity. RoMoMatter will contribute to the value of the reproductive justice approach to unravel the harmful role that anti-gypsyism, sexism, violence, and poverty play in Roma girls’ quest to feel recognized and influential in their social spheres [14]. In recent years, interest in the reproductive aspects of Roma women has grown dramatically, but there are very few initiatives focused on building the political agency of Roma girls to make free decisions about their identities, their bodies, their families, and communities. This is a key feature of the growing global interest to address gender oppression as a priority area in the post Decade of Roma Inclusion (after 2015) agenda [30,47].

The reproductive justice approach overcomes the limitations focused on sexual and reproductive health oriented to addressing issues such as virginity, contraception and birth control [48]. Conversely, this approach focuses on ensuring that Roma women acquire—during their childhood and adolescence—the symbolic, psychosocial, relational and community resources to make decisions about their lives and build their life goals freely. The ultimate target is to transform reproduction into a source of liberation rather than reproducing inequities [33]. Moreover, reproductive justice observes the inequalities suffered by Roma women linked to the intersection of marginalizing identities (gender, ethnicity, class) that are linked to a distribution of power that threatens their self-determination by perpetuating norms, strategies, and legislation aimed at sexual and social control of women [49]. Illuminating women’s reproductive experiences, reproductive justice helps Roma girls to balance traditional and survival values that exist within their communities (such as the relevance of religion or traditional family values) with secular-rational and self-expression values (i.e., motivation to participate in decision-making in economic and political life), which allow girls to visualize multiple and different aspirations for their lives [50].

To this end, RoMoMatter seeks to consolidate the scope of studies that combine an intersectional and developmental lens in the study of Roma women and girls’ wellbeing. RoMoMatter will promote empowering community settings in which girls will identify the multiple ways in which oppressive structural mechanisms transform their development contexts (e.g., family, school, social networks, communities, institutions) in sources of experiences of violence, poverty, stress, discrimination, and prejudice [27,51,52,53]. Linking the psychosocial development of Roma girls to reproductive justice, RoMoMatter hopes to forge new ways of building ethnicity, race, gender, class, as well as a new practice based on social justice for Roma people [54].

RoMoMatter will also contribute to the development of methodologies that generate spaces of collaboration between scholars, civil organizations, policymakers, and activists led by Roma women and girls to advocate for the control of their bodies and their lives. Traditionally, Roma women—more so girls and adolescents—have occupied a peripheral position in the different stages of research and action processes, despite repeated calls to increase their leadership as co-researchers and allies [37,41,55,56]. The methodological approach inspired by reproductive justice allows us to confront a system of knowledge generation that continually pathologizes divergent voices and violates their rights in order to maintain oppressive conditions. If our questions, objectives, methods, results, and dissemination do not confront the status-quo, our research activity risks reproducing the same inequities, ultimately blaming victims for their suffering rather than generating liberating knowledge capable of overcoming them [57].

The multiple methodologies that are employed in RoMoMatter (e.g., community mapping, narratives, Photovoice) aim to highlight that overcoming the inequities suffered by Roma women will come from initiatives that promote scientific evidence obtained as a product of their joint vision of inequalities [58]. The multiple voices in the collection of evidence highlight what works, for whom, and in what circumstances. In this process, communities must have undisputed leadership, because it is rightfully theirs [37]. Lastly, overcoming the inequities suffered by Roma girls requires processes of accountability that empower communities to achieve effective results [59]. This requires evaluation processes that respect democratic rights and freedoms. Because the inequities suffered by Roma girls are multiple, intertwined, and complex, all key stakeholders must leave their comfort zones to achieve a common discourse and share objectives [60], establish alliances and partnerships based on trust and respect, and take action for which they are responsible [37].

### Limitations

However, implementing these methodologies entails some risks, such as the difficulties of engaging RoMoMatteR’s participants throughout the project (i.e., low availability of Roma girls to participate, low involvement of local coalition members or scarce willingness of significant others—especially parents—to support the project). This issue is related to the difficulties to adapt the project activities and materials to each context’s needs and realities, as the actions will be implemented in four different Roma local contexts. Moreover, as RoMoMatteR includes advocacy actions to be developed, we could face some opposition from the wider community to agree on and embrace Roma girls’ recommendations. Lastly, the changing social conditions and needs (especially in COVID-19 times) entail a delay in delivering activities, affecting the timeline of the project and therefore, emphasizing the limitations mentioned before.

## 4. Conclusions

RoMoMatter is focused on contributing to the main goals of the European Roma Framework for National Roma Integration Strategies concerning girls and women, who are one of the most vulnerable sectors of Roma in all countries. Building community and promoting liberating narratives among Roma girls contribute to improving their educational aspirations and achievements, their employability in higher and more influential positions, their social status, the shared upbringing of children, equitable family structure, and couple relationships, while respecting and enhancing Roma values. At a technical–scientific level, RoMoMatteR will generate evidence of a conceptual and methodological model to transform values in Roma girls and adolescents at risk of social exclusion. Roma women are one of the central axes of many scientific initiatives since the persistent failure of previous endeavours aimed at improving their social inclusion in European countries. RoMoMatteR aims to impact the elements identified as determinants of this failure: dismantling institutional discrimination, promoting professional competence, cross-sectoral actions, and working with minors and empowering them to generate changes from the population itself. RoMoMatteR focuses directly on the United Nations’ sustainable development goals for gender equality and seeks to eliminate all forms of violence against girls at family and community levels.

## Figures and Tables

**Table 1:** Framing Roma girl Participatory Action Research to empower mattering linked to reproductive justice.

Goal	Procedure
1st Component: Building local coalitions	
Facilitate understanding; create trust and a collaborative environment to assure sustainability.	Field Work Groups (Research–Community Partnership) will identify and recruit a core group of local key stakeholders from the community (10–15 members).
2nd Component: Mapping community assets	
Identify community initiatives and programs relevant to the Roma girls and women	Desk reviews of plans and programs at national, regional and local level. Interviews with key stakeholders from the community who implement/adapt non-institutionalized actions not included in the desk reviews.
3rd Component: Mapping Roma women narratives	
Collect personal and collective narratives from Roma women about their mattering linked to reproductive justice	Conducting individual interviews and focus groups (at least 10 narratives in each study context).
4th Component: Mapping scientific evidences	
Explore the indexed scientific literature on Roma Girl’s Participatory Action Research to empower mattering linked to reproductive justice.	The scoping reviews followed the protocols proposed by Arksey and O’Malley [38] and Colquhoun et al. [39]
5th Component: Toolbox for Roma Girls Participatory Action Research	
Create an on-line toolbox available at the project website.	Gathering the results of the different mapping activities carried out previously.

**Table 2:** Implementation of the Roma girls’ Participatory Action Research: Activities and procedures.

Activities	Procedure
1st Stage: Training and building cohesion among Roma girls’ families and significant adults	
(1) Training facilitators	A training package for facilitators to implement and evaluate the activities will be developed following the recommendations and guidelines provided in the Community Tool Box ^1^.
(2) Building cohesion among participants	Monthly meetings in informal settings in order to build collaboration, ensure implementation, and reflect on the process, conducting meaningful conversations that matter aimed at listening to the community interests, needs, and strengths while researchers relate to them as partners [41].
2nd Stage: Developing critical thinking on reproductive justice through Photovoice	
(3) Developing girls’ capacity to critically think in their aspirations for the future	Girls are provided with a camera and trained to respond through photographs to the question “What are your dreams for the future?”
	Girls interact with women models; take pictures to envision aspirations on becoming women.
(4) Developing capacity to articulate and communicate their narratives of aspirations	Girls play games related to categorization and then, group and label their photos.
	Girls reflect individually on the photographs taken with the assistance of a facilitator, articulate their own narratives and discuss them in small groups
(5) Disseminating their aspirations	Girls carry out local activities to spread their aspirations with the support and participation from community coalition members
3rd Stage: Building capacity of Roma girls to advocate for their aspirations	
(6) Developing Roma girls’ capacity to identify resources to make your aspirations a reality	The girls are invited to respond through photographs to the question “What resources do you have and what resources do you need to make your aspirations came true?”
(7) Developing recommendations and an advocacy plan	Girls take photos in their communities and home which reflect the resources they have and the resources they need to make their aspiration come true
	Girls sort and label photos, reflect individually with the assistance of facilitator and then have a small group discussion
	All girls develop a list of recommendations and agree through consensus an advocacy plan to share their recommendations
(8) Disseminating their recommendations and advocacy plan in community exhibitions	Girls carry out local level advocacy activities with the support and participation from community coalition members

^1^https://ctb.ku.edu/en/table-of-contents/leadership/group-facilitation.

**Table 3:** Building evaluation capacity among girls and their communities; Process, implementation and outcome components.

Goals	Indicators
Process evaluation:	
Degree to which all activities planned are carried out.	Timeline. Resources and structures. Barriers to implementation. Strategies are created to prevent and/or overcome identified barriers. Monitoring process is established. Recruitment processes established
Implementation evaluation:	
Assessing if activities were implemented as planned, as well as the strengths and challenges.	Roma Girls participation in actions and satisfaction with the process Facilitators commitment to actions. Degree to which RGPAR and Empowerment Evaluation training meet the needs of facilitators. Community involvement and support for the initiative. Cocreation approaches promoted and applied.
Outcomes evaluation.	
Through a pretest–posttest with comparation group design, the results and consequences of RGPAR among participants will be assessed.	Roma Girls expand their personal networks Roma Girls change their narratives and envision their futures Changes on Roma girls’ life satisfaction Increased community support for Roma girls

**Figure A1:**
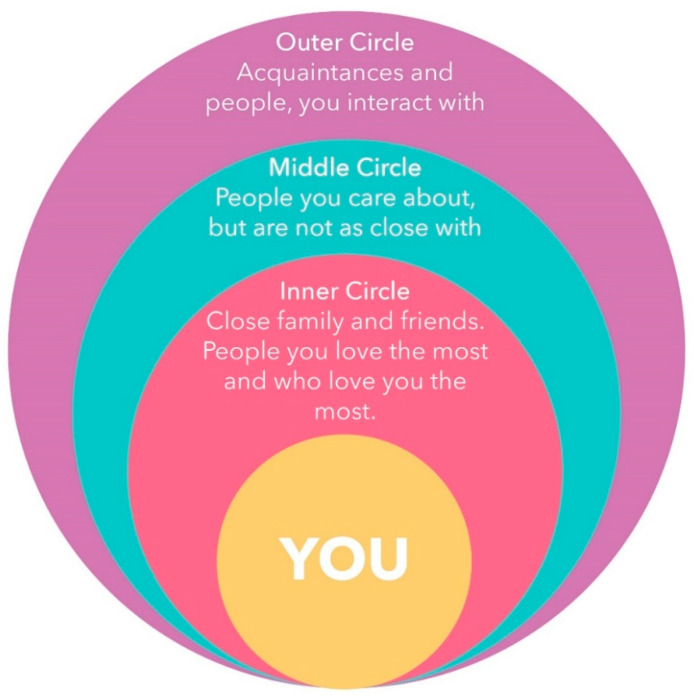
Social Convoy Activity [43].

## Appendix A. Data Collection: Interview Questionnaires for Roma Girls

**Table A1:** Relevance of identity roles scale.

How Important to You in Obtaining Your Life Goals Is…	Not at All	Slightly	Generally	Very/Completely
…finishing high school	1	2	3	4
…going to college	1	2	3	4
…being a mother	1	2	3	4
…getting married	1	2	3	4
…starting a family	1	2	3	4
…having a satisfactory job	1	2	3	4
…having a close relationship with your friends	1	2	3	4
…having a close relationship with your birth family	1	2	3	4

**Table A2:** Life satisfaction scale [45].

Please, Rate the Following Sentences with the Rank:	Not at All	Slightly	Generally	Very /Completely
In most ways my life is close to ideal.	1	2	3	4
The conditions of my life are excellent.	1	2	3	4
I am satisfied with my life.	1	2	3	4
So far, I have gotten the important things I want in life	1	2	3	4
If I could live my life over, I would change almost nothing.	1	2	3	4

**Table A3:** Mattering questionnaire: family, school and community level [15].

Please, Rate the Following Sentences with the Rank:	Not at All	Slightly	Generally	Very /Completely
The people in my family value me as a person.	1	2	3	4
I feel I help meet the needs of my family.	1	2	3	4
I am an important part of my family.	1	2	3	4
My family would not be the same without me.	1	2	3	4
I influence the lives of people in my family.	1	2	3	4
I feel like I matter to my family.	1	2	3	4
My ideas are valued by the people in my family.	1	2	3	4
I am appreciated by the people in my family.	1	2	3	4
I have an influence on my family.	1	2	3	4
The people in my school value me as a person.	1	2	3	4
I feel I help meet the needs of my school.	1	2	3	4
I am an important part of my school.	1	2	3	4
My school would not be the same without me.	1	2	3	4
I influence the lives of people in my school.	1	2	3	4
I feel like I matter to people in my school.	1	2	3	4
My ideas are valued by the people in my school.	1	2	3	4
I am appreciated by the people in my school.	1	2	3	4
I have an influence on the way my school is.	1	2	3	4
The people in my community value me as a person.	1	2	3	4
I feel I help meet the needs of my community.	1	2	3	4
I am an important part of my community.	1	2	3	4
My community would not be the same without me.	1	2	3	4
I influence the lives of people in my community.	1	2	3	4
I feel like I matter to my community.	1	2	3	4
My ideas are valued by the people in my community.	1	2	3	4
I am appreciated by the people in my community.	1	2	3	4
I have an influence on my community.	1	2	3	4

**Table A4:** Socio-political agency scale [44].

Please, Rate the Following Sentences with the Rank:	Not at All	Slightly	Generally	Very /Completely
Youth like me can really understand what’s really going on with my community	1	2	3	4
Youth like me have the ability to participate effectively in community activities and decision making	1	2	3	4
There are plenty of ways for people like me to have a say in what our government does	1	2	3	4
Most of community leaders would listen to me	1	2	3	4
